# Clock-hour topography and extent of outer retinal damage in hydroxychloroquine retinopathy

**DOI:** 10.1038/s41598-022-15217-3

**Published:** 2022-07-12

**Authors:** Ko Eun Kim, Ji Hong Kim, Young Hwan Kim, Seong Joon Ahn

**Affiliations:** 1 Department of Ophthalmology, Asan Medical Center, Ulsan University College of Medicine, Seoul, Republic of Korea; 2 Department of Ophthalmology, Hanyang University Hospital, Hanyang University College of Medicine, 222-1 Wangsimni-ro, Seongdong-gu, Seoul, 04763 Republic of Korea

**Keywords:** Retinal diseases, Medical imaging

## Abstract

In this study, we investigated the clock-hour topographic characteristics and extent of photoreceptor and retinal pigment epithelium (RPE) damage and correlated the extent with functional defects in eyes with hydroxychloroquine retinopathy. A total of 146 eyes of 75 patients diagnosed with hydroxychloroquine retinopathy were included. The clock-hour topographic characteristics (relative to the fovea) and extent of the photoreceptor and RPE defects in the parafoveal and pericentral areas were evaluated by reviewing the radial-scan optical coherence tomography (OCT) and wide-field fundus autofluorescence (FAF) images. The extent of outer retinal damage in the parafoveal and pericentral areas were correlated with the perimetric parameters of the Humphrey 10–2 and 30–2 tests, respectively. Although the photoreceptor damage was most commonly noted at the temporal to inferior locations in both parafoveal and pericentral areas, the RPE damage in the pericentral eyes was most commonly noted in the nasal area and showed topographic discrepancies with photoreceptor damage. The extent of RPE damage was almost identical between OCT and FAF images, whereas photoreceptor defect extent was significantly greater on OCT images. The extent of parafoveal and pericentral photoreceptor damage on OCT images was significantly correlated with perimetric parameters of the 10–2 and 30–2 tests, respectively (all *P* < 0.05). Our findings on the detailed topographic characteristics using a clock-hour-based system and significant correlation between the structural extent and perimetric parameters suggest that this evaluation may facilitate more comprehensive descriptions of structural damage extent and predictions of visual function.

## Introduction

Hydroxychloroquine retinopathy is well-recognized toxic retinopathy occurring in long-term users of hydroxychloroquine, which is widely used for treating diverse rheumatologic and dermatologic diseases such as systemic lupus erythematosus (SLE) and rheumatoid arthritis (RA)^1–3^. The retinopathy is characterized by outer retinal damage, typically the defects on the photoreceptors and/or retinal pigment epithelium (RPE). According to the recent reports using modern screening modalities^4^, the prevalence of hydroxychloroquine retinopathy is estimated to be approximately 5–10% among long-term users, which is significantly higher than previously estimated^5,6^. With the increasing number of the population using the medication^7^, the number of patients with retinal toxicity is expected to grow.

The classification of hydroxychloroquine retinopathy is based on retinopathy severity, currently as early (patchy photoreceptor damage), moderate (photoreceptor damage of > 180° degree without RPE defects), and severe (combined RPE damage). This is clinically important as the information can be used to predict the future behavior of retinopathy following drug cessation^8–10^. However, the distinction between early and moderate retinopathy is sometimes ambiguous and the modalities for disease severity stratification are lacking consensus. Moreover, the extent can be more detailed than simply documenting early (localized) or moderate (over 180 degrees) diseases. Further, the discrimination between moderate and severe stages is based on the presence of RPE defects and does not reflect the extent of the involved areas. Accordingly, some moderate cases may have larger areas of photoreceptor damage and thus, greater functional defects than severe cases.

Topographically, the progression pattern of hydroxychloroquine retinopathy can be divided into circumferential and centripetal/centrifugal^9^. A recent report showed that circumferential progression was dominant in earlier stages, while both circumferential and centripetal enlargement were significant in later stages in eyes with hydroxychloroquine retinopathy^9^. This may explain why the lesions in eyes with hydroxychloroquine retinopathy have a partial- or full-ring shape, and the evaluation of retinopathy extent and progression requires an approach representative of the circumferential extent of retinal damage, which may be utilized to classify the eyes into severity groups. Nevertheless, several studies of structural progression have emphasized advancement of retinal damage toward the fovea as the criterion for structural progression^10,11^.

Based on the limitations of the current classification and recent findings on hydroxychloroquine retinopathy, we hypothesized that the clock-hour-based evaluation of topographic extent and location may be useful for determining retinopathy extent and correlating it with functional defects in hydroxychloroquine retinopathy. By performing separate analyses of parafoveal and pericentral damage, we aimed to evaluate the clock-hour extent and topographic characteristics of photoreceptor and RPE damage in these two areas affected by hydroxychloroquine retinopathy. Furthermore, we compared the extent of damage evident on OCT and FAF images to address the modalities used to evaluate retinopathy extent.

## Methods

### Patients

The patients were retrospectively included from a patient cohort of hydroxychloroquine retinopathy comprising 79 diagnosed with retinopathy at Hanyang University Hospital between January 1, 2015 and December 31, 2021. Among the 158 eyes of 79 patients, those with coexistent retinal or macular diseases affecting the outer retina (e.g. age-related macular degeneration, central serous chorioretinopathy, and retinal vascular diseases; n = 9 eyes) and those with the poor image quality of OCT or FAF disturbing image analyses (n = 3 eyes) were excluded. Finally, 146 eyes from 75 patients were included in our analyses. This study was approved by the ethics committee and the Institutional Review Board of Hanyang University Hospital and followed the tenets of the Declaration of Helsinki. Informed consent was waived due to the retrospective design of the study.

### Examination

All patients underwent comprehensive ophthalmologic examinations at baseline and follow-up visits, including best-corrected visual acuity assessment, noncontact tonometry, automated refraction, slit-lamp examination, and dilated fundus examination. Swept source OCT (DRI-OCT Triton; Topcon Inc., Tokyo, Japan) and FAF (Optos 200Tx; Optos PLC, Dunfermline, United Kingdom) were performed to detect structural damage in all the patients. To evaluate functional deterioration, standard automated perimetry using the 30–2, 10–2, or both strategies (Humphrey Field Analyzer II or III; Carl Zeiss Meditec, Dublin, CA) was performed.

### Evaluation

According to the latest guidelines of the American Academy of Ophthalmology, the diagnosis of hydroxychloroquine retinopathy was made when the abnormal findings from at least one objective test (OCT and FAF) were detected, confirming subjective test abnormality^4^. OCT and FAF findings included photoreceptor and/or RPE defects and hyper- or hypoautofluorescence in the parafoveal or pericentral areas, respectively^4,12–14^.

As in previous studies, we classified hydroxychloroquine retinopathy into parafoveal (outer retinal defects in area 2–6° from the fovea), pericentral (defects in the area > 6° from the fovea), or mixed (both patterns) retinopathy according to disease pattern (location)^4,9,13,15,16^. Furthermore, according to disease severity, retinopathy was divided into early (patchy or localized photoreceptor defects), moderate (photoreceptor defects greater than 180° in extent without RPE involvement), and severe (outer retinal defects with RPE involvement) retinopathy^7,9–12^.

**Figure 1:**
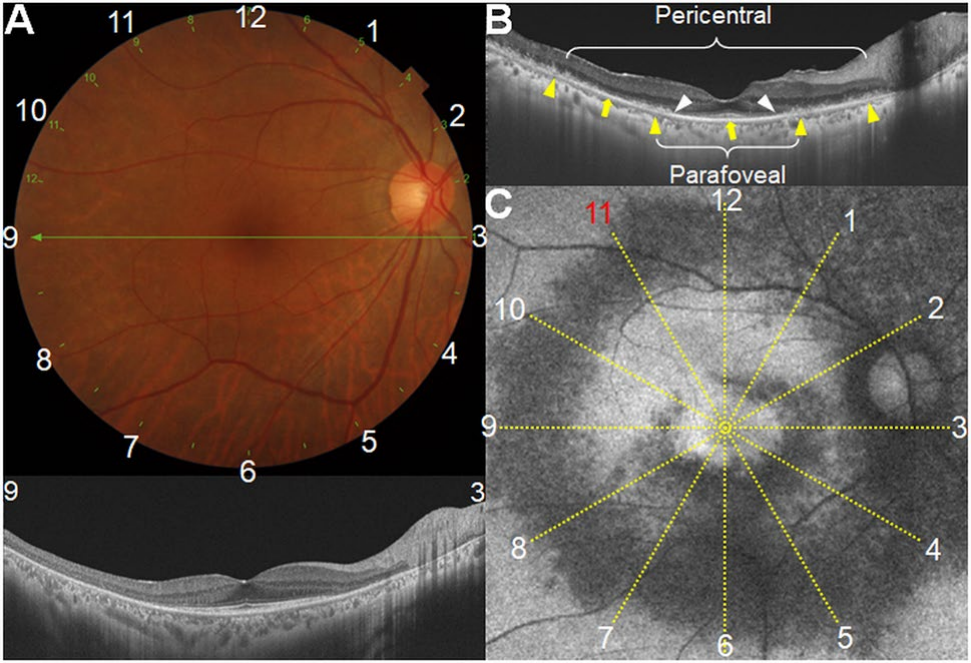
Representative images for evaluating clock-hour topography and outer retinal damage (white and yellow arrowheads) extent in eyes with hydroxychloroquine retinopathy. (**A**) Fundus photograph (top) showing the scanning area of the optical coherence tomography (OCT) scans used in this study (12-radial scan). Six radial intersecting line scans, each at 30-degree intervals, were used to scan each clock hour of the retina (**B**). (**C**) Fundus autofluorescence (FAF) images of an eye with severe hydroxychloroquine retinopathy of combined parafoveal and pericentral involvement. Compared to the relatively preserved retinal pigment epithelium (RPE) line (arrows), the parafoveal and pericentral areas showed thinning and attenuation of the RPE line (yellow arrowheads). FAF image of the eye merged with a 12-line grid radiating from the foveal center indicating ring-shaped parafoveal and pericentral lesions that spare only an 11 clock-hour area (red text) in the parafovea.

The clock-hour topographic characteristics and extent of outer retinal disruption were evaluated by reviewing the 12-radial OCT scans, which produced 12-radially oriented line scans passing through the fovea (Fig. 1) with a 12-mm scan length. Among the 12-line scan images, six images covering 1 to 12 clock-hour retinal areas (white texts in Fig. 1) were reviewed. Photoreceptor damage on OCT (white arrowheads in Fig. 1) was determined as loss or disruption of the ellipsoid zone, whereas RPE damage (yellow arrowheads in Fig. 1) was defined as the thinned and attenuated RPE/Bruch’s membrane complex line. For each clock-hour area, each photoreceptor and RPE damage was evaluated from the baseline and follow-up OCT images. For those with both parafoveal and pericentral damage, clock-hour involvement was separately analyzed for each damage (Fig. 1B).

To facilitate the determination of clock-hour involvement of retinal damage on FAF, reference clock hour lines, consisting of 12-radial straight lines diverging from the fovea, were merged with the FAF images (Fig. 1C). For each clock hour location, the presence of abnormal FAF findings, either hyperautofluoresence or hypoautofluoresence, was evaluated. Hyper- and hypo-autofluorescence were evaluated separately for those with FAF abnormalities. Two investigators (S.J.A. and K.E.K.), who were blinded to the clinical information, independently evaluated the photoreceptor and RPE damage on OCT and hyper- and hypoautofluorescence on FAF images. Any discrepancy in the judgment of photoreceptor and RPE involvement between the two investigators was resolved through discussion. The data in the left-eye images were converted to the right-eye format by matching identical test locations, leading to one data for each clock hour location (e.g. 11 and 10 clock hours [superonasal areas] in the left eye converted to 1 and 2 clock hour counterparts, respectively).

To evaluate the functional correlation with structural damage, quantitative perimetric parameters such as mean deviation (MD) and pattern standard deviation (PSD) of Humphrey 10–2 and 30–2 tests and visual field index (VFI) of Humphrey 30–2 were used.

### Analysis

Descriptive statistics were used for patient demographics and clinical characteristics including patterns and severity of retinopathy and clock-hour topographic information of retinopathy. All continuous variables were presented as mean ± standard deviation. The extent of photoreceptor and RPE damage was compared between the imaging modalities. Pearson correlation analyses were performed to evaluate the relationships between the clock hour extents of photoreceptor and RPE damage and the perimetric parameters in all the patients and in subgroups separated based on severity (early, moderate, and severe) and pattern of retinopathy. Statistical significance was set at *P* < 0.05. All statistical analyses were performed using SPSS software version 23 (IBM Corp., Armonk, NY, USA).

## Results

### Patient demographics and clinical characteristics

The demographic and clinical characteristics of the included patients, comprising 72 women and three men, are presented in Table 1. The patients had SLE in 36 (48.0%), RA in 34 (45.3%), or other rheumatologic diseases (5, 6.7%), such as connective tissue diseases and Sjogren’s syndrome, for which hydroxychloroquine was used for the treatment. The mean age was 55.9 ± 13.6 years and the duration of the hydroxychloroquine use was 14.7 ± 7.1 years on average. Among the 146 eyes, 27 (18.5%), 89 (61.0%), and 30 (20.5%) eyes had parafoveal, pericentral, and mixed retinopathy, respectively. The severe stage was the most common (n = 58, 39.7%), followed by early (n = 49, 33.6%) and moderate (n = 39, 26.7%) stages. The mean daily dose was 251.1 mg in patients with an average daily hydroxychloroquine dose to body weight ratio of 4.9 ± 1.4 mg/kg.

**Table 1:** Demographic data and clinical characteristics of the 75 patients included in this study.

Characteristics	Results
Sex, female (%)	72 (96)
Age, years (range)	55.9 ± 13.6 (20–78)
Diagnosis, SLE: RA: others* (%)	36 (48): 34 (45.3): 5 (6.7)
Severity of retinopathy, early: moderate: severe	49 (33.6): 39 (26.7): 58 (39.7)
Pattern of retinopathy, parafoveal: pericentral: mixed	27 (18.5): 89 (61.0): 30 (20.5)
Mean daily dose, mg (range)	251.1 ± 72.6 (100–400)
Mean daily dose/real body weight, mg/kg (range)	4.9 ± 1.4 (1.9–8.5)
Mean duration of hydroxychloroquine use, yrs (range)	14.7 ± 7.1 (1–33)
Mean cumulative dose, g (range)	1340.4 ± 707.3 (73–3066)
Mean cumulative dose/real body weight, g/kg (range)	26.0 ± 15.1 (1.4–68.4)

*RA* rheumatoid arthritis, *SLE* systemic lupus erythematosus.

*Other medical indications included mixed connective tissue disease and Sjögren syndrome.

### Clock-hour topography of outer retinal damage on OCT and FAF

**Figure 2:**
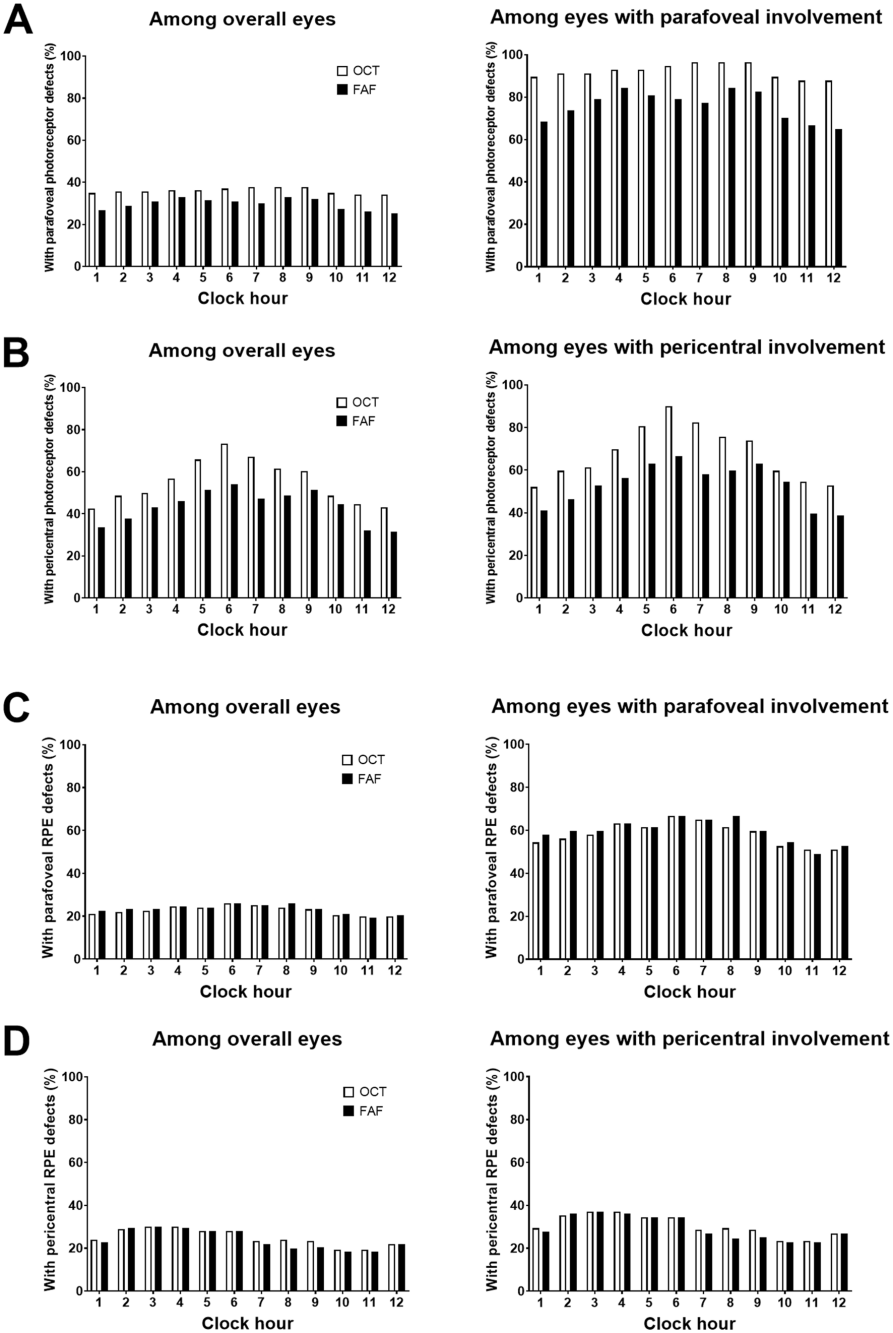
(**A**) Percentages of eyes with photoreceptor defects on optical coherence tomography (OCT) and hyper- or hypoautofluorescence on fundus autofluorescence (FAF) images on the parafoveal area among the overall eyes (left) and those with parafoveal involvement (right). (**B**) Those with pericentral photoreceptor defects on OCT and hyper- or hypoautofluorescence among the overall (left) and pericentrally involved eyes (right). (**C**, **D**) indicate parafoveal and pericentral retinal pigment epithelium defects on OCT and FAF (as hypoautofluorescence) images among overall eyes (left) and those with parafoveal or pericentral involvement (right).

Figure 2 shows the percentages of eyes with photoreceptor defects on OCT scans and those with abnormal findings on FAF imaging, hyper- or hypo-autofluorescence (indicating photoreceptor defects with or without RPE defects) for each clock-hour location in each parafoveal (Fig. 2A) and pericentral (Fig. 2B) areas, respectively. Using the clock-hour based topography of the retinal damage, the most common location for parafoveal photoreceptor damage was the temporal (9 o’clock) to inferior (6 o’clock) areas. Pericentral damage was most commonly noted at 6 o’clock, followed by 7, 5, 8, and 9 o’clock, which showed a similar distribution to parafoveal ones (inferior to temporal). Superior locations (from 11 to 1 o’clock) were the least common areas for both parafoveal and pericentral photoreceptor damage. Subgroup analyses in early retinopathy showed similar tendency that early damage, both parafoveal and pericentral ones, were mostly in the temporal and inferior areas (Supplemental Fig. S1).

The percentages of photoreceptor defects between OCT and FAF imaging in Fig. 2 demonstrate that the percentage of eyes with photoreceptor damage at each clock-hour location on OCT was greater than that with any abnormality on FAF for both parafoveal and pericentral areas. This indicates the better sensitivity of OCT over FAF for the detection of photoreceptor defects in the parafoveal or pericentral areas. Moreover, the percentage of photoreceptor defect in each clock-hour location was remarkably different between FAF and OCT in those with early pericentral disease except superior (10 to 2 o’clock) locations (Supplemental Fig. S1), in which early cases of either pattern rarely had photoreceptor damage.

**Figure 3:**
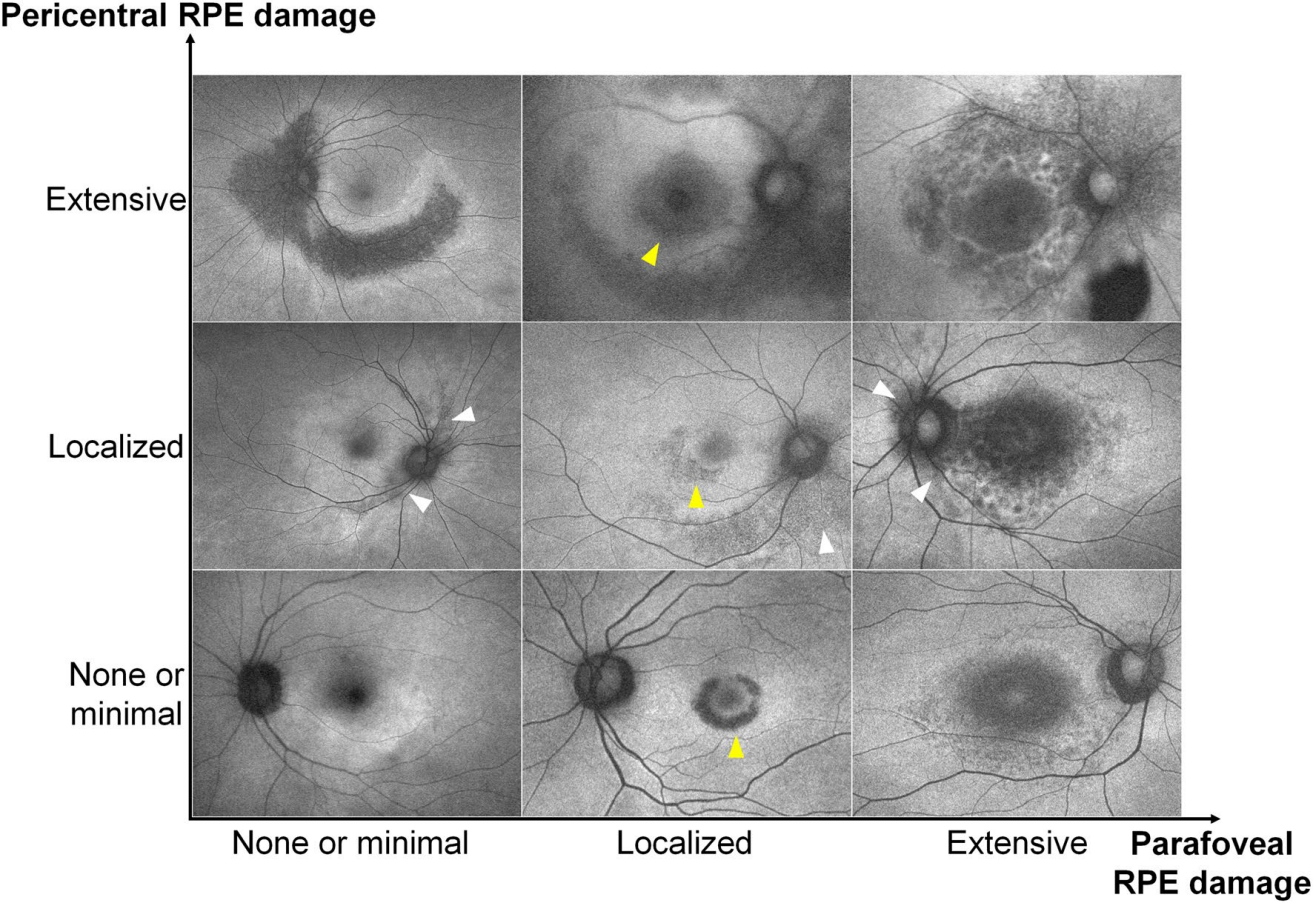
Representative cases of hydroxychloroquine retinopathy separated by the degrees of parafoveal (x-axis) and pericentral (y-axis) retinal pigment epithelium (RPE) defects. Parafoveal and pericentral RPE defects are commonly noted on the inferior parafoveal (yellow arrowheads) and peripapillary (nasal, white arrowheads) areas, respectively.

Figure 2C and D illustrate the percentages of eyes with RPE defects on OCT scans and those with hypoautofluorescence on FAF for each clock-hour location in the parafoveal and pericentral areas, respectively. Interestingly, the most common locations for parafoveal and pericentral RPE damage were different. For example, parafoveal RPE damage was most commonly noted at 6 o’clock (followed by 8 and 7 o’clock), whereas pericentral RPE damage was most common at 3 o’clock (followed by 4 and 2 o’clock). As shown in Fig. 3, the representative cases of parafoveal and pericentral retinopathy showed RPE defects initially or commonly in the inferior parafoveal and peripapillary (nasal) areas, respectively.

### Clock-hour extent of photoreceptor and RPE damage in eyes with hydroxychloroquine retinopathy

The clock-hour extents of photoreceptor and RPE damage on OCT and FAF imaging are presented in Table 2. The number of involved clock-hour areas for parafoveal photoreceptor damage on OCT and FAF were 4.0 ± 5.6 and 3.3 ± 5.1, respectively, among the overall eyes and 11.2 ± 2.2 and 9.1 ± 4.3, respectively, among those with parafoveal damage, respectively, showing significant differences (both *P* < 0.001). The differences in evident pericentral damage area between OCT (6.2) and FAF (4.9) images were also significant (*P* < 0.001). However, those of the RPE defects on OCT and FAF images were comparable on parafoveal (2.6 and 2.6, respectively) and pericentral (2.8 and 2.7, respectively), showing no statistically significant difference between the two modalities (both *P* > 0.05). Subgroup analyses revealed that the difference between the OCT and FAF images was remarkable in eyes with both early parafoveal (5.4 vs. 2.6; *P* = 0.019) and pericentral (3.1 vs. 0.8; *P* < 0.001) retinopathies.

**Table 2:** Clock hour extents of photoreceptor and retinal pigment epithelium (RPE) damages on parafoveal and pericentral areas among those with parafoveal (n = 57 eyes) and pericentral involvement (n = 119 eyes) on optical coherence tomography (OCT) or fundus autofluorescence (FAF).

Parameters	Overall eyes (n = 146)			Parafoveal involvement (n = 57)			Pericentral involvement (n = 119)		
	OCT	FAF	P value*	OCT	FAF	P value*	OCT	FAF	P value*
**Photoreceptor defects**									
Parafoveal, hrs	4.0 ± 5.6	3.3 ± 5.1	** < 0.001**	11.2 ± 2.2	9.1 ± 4.3	** < 0.001**	N/A	N/A	N/A
Pericentral, h	6.2 ± 5.1	4.9 ± 5.3	** < 0.001**	N/A	N/A	N/A	8.1 ± 4.4	6.4 ± 5.2	** < 0.001**
**RPE defects**									
Parafoveal, h	2.6 ± 4.7	2.6 ± 4.7	0.234	7.0 ± 5.3	7.2 ± 5.3	0.237	N/A	N/A	N/A
Pericentral, h	2.8 ± 4.6	2.7 ± 4.5	0.259	N/A	N/A	N/A	3.7 ± 5.0	3.6 ± 4.9	0.260

Significant values are in bold.

*Paired t test.

*N/A* not applicable.

### Correlation between the clock-hour extent of retinal damage and perimetric function

**Figure 4:**
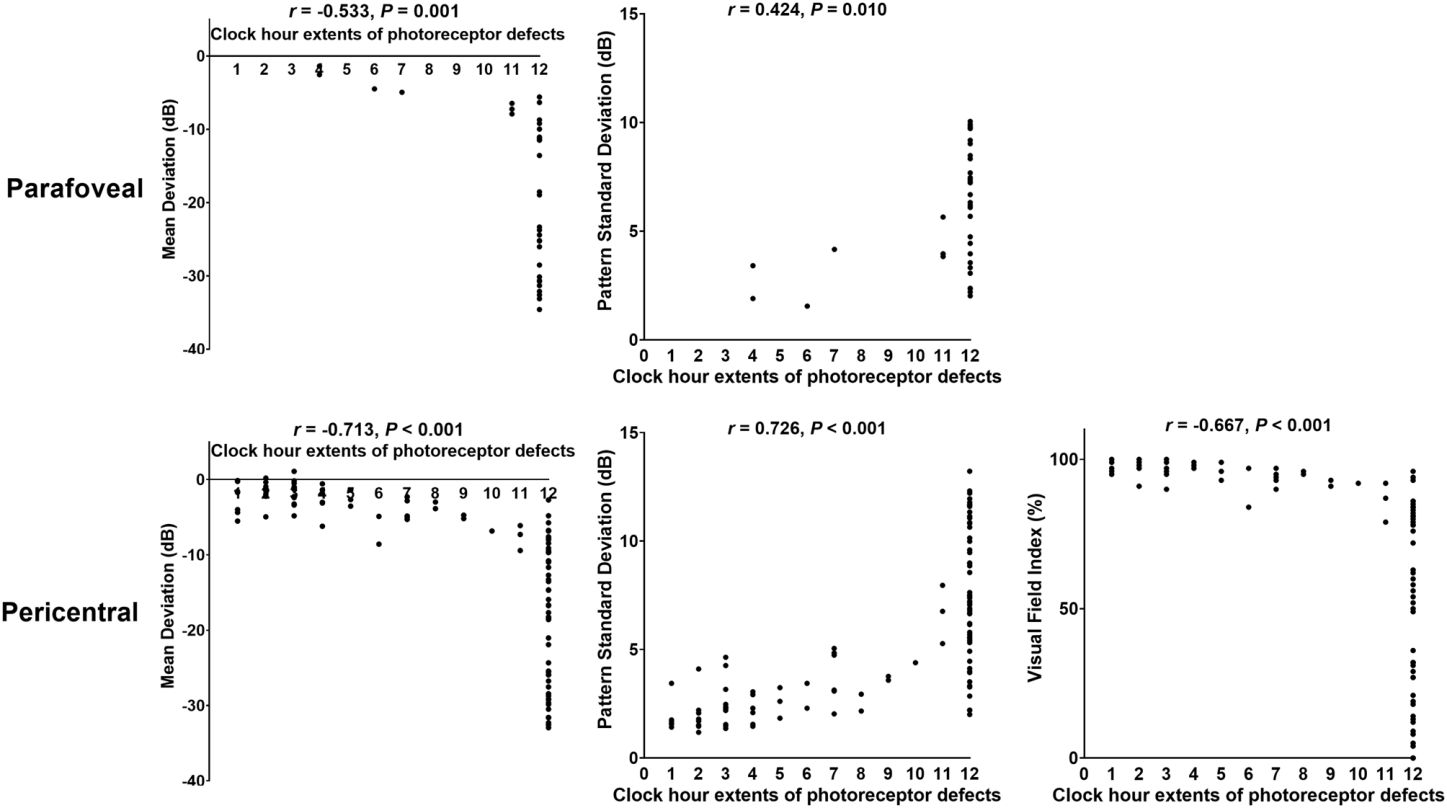
Correlation between clock-hour extents of parafoveal photoreceptor defects and mean deviation (MD) and pattern standard deviation (PSD) on the Humphrey 10–2 test (top) and that between those of pericentral photoreceptor defects and MD, PSD, and visual field index on the 30–2 test (bottom).

Figure 4 shows the correlations between perimetric parameters on the 10–2 and 30–2 tests and clock-hour extents of photoreceptor damage on OCT images among the eyes with parafoveal (top) and pericentral (bottom) damage. MD and PSD on the 10–2 tests significantly correlated with the clock-hour extents of parafoveal damage (r = − 0.533 and 0.424, *P* = 0.001 and 0.010 for MD and PSD, respectively). Those on the 30–2 tests showed significant correlations with the pericentral clock-hour extents of the photoreceptor defects (all *P* < 0.05). For RPE damage, MD and PSD on the 10–2 test were significantly correlated with the parafoveal RPE damage extent, whereas PSD on the 30–2 test showed an insignificant correlation with pericentral damage (Supplemental Fig. S2).

**Figure 5:**
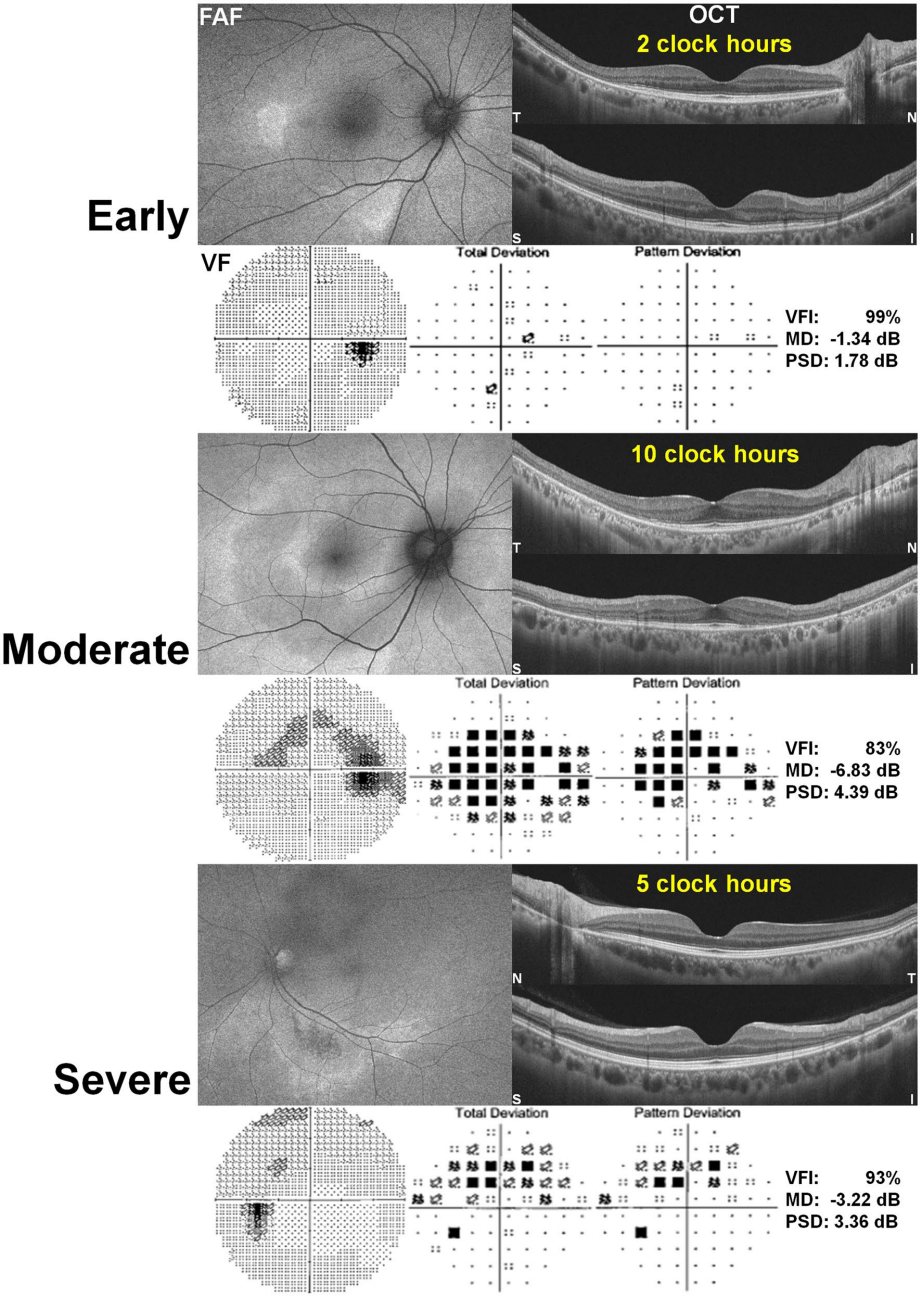
Photographic examples of fundus autofluorescence (FAF), optical coherence tomography (OCT), and automated visual field (VF) results including visual field index (VFI), mean deviation (MD), and pattern standard deviation (PSD) in eyes with early, moderate, and severe retinopathy. The extent of photoreceptor damage on OCT (indicated in yellow) showed 2, 10, and 5 clock-hour extents for the respective cases of early, moderate, and severe retinopathy. The moderate retinopathy case showed larger clock-hour involvement of retinal damage than the severe one, also showing worse perimetric function, represented by worse VFI, MD, and PSD. T = temporal; N = nasal; S = superior; I = inferior.

**Figure 6:**
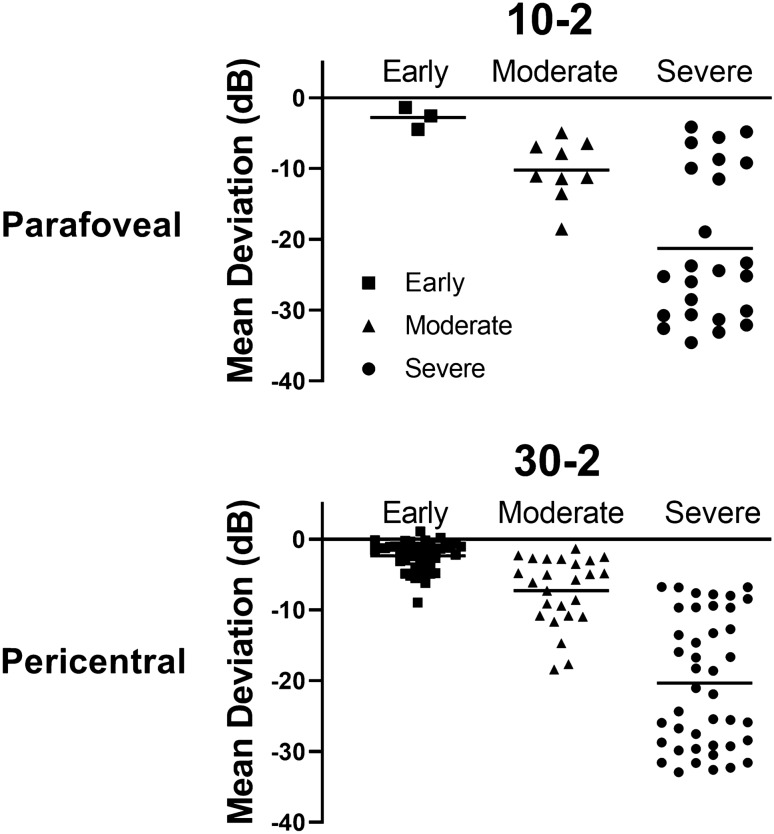
Mean deviation (MD) values on Humphrey 10–2 and 30–2 visual field tests in eyes with parafoveal and pericentral involvement, respectively, separated into severity groups according to the current three-stage (early, moderate, and severe) classification. The horizontal bars in each severity group indicate the mean values of MD. In both parafoveal and pericentral retinopathies, the mean MD values showed decreasing trend according to the disease severity. However, in moderate and severe retinopathies, the MD values were scattered and distributed in a wide range and this was more evident in severe retinopathy.

Figure 5 shows photographic examples of eyes with early, moderate, and severe retinopathy and the extents of photoreceptor damage on OCT and FAF in the eyes. The eyes with severe retinopathy showed a smaller number of clock-hour extent of photoreceptor damage on OCT or FAF, less extensive scotoma on pattern deviation plot, and greater MD and VFI of 30–2 visual fields than those with moderate retinopathy, indicating more preserved visual function in severe eyes. There were remarkable differences in the mean value of MD (horizontal line) among the three stages (Fig. 6). However, eyes with severe disease showed a wide range of MD compared to those with early or moderate disease and several eyes with severe disease showed greater MD values than eyes with moderate disease.

## Discussion

The present study demonstrated the clock-hour topography and extent of photoreceptor and RPE damage in hydroxychloroquine retinopathy by separate analyses of parafoveal and pericentral damage. From the analyses, this study revealed the topographical characteristics of hydroxychloroquine retinopathy and discrepancies in the distribution between the photoreceptor and RPE damage in the pericentral areas. Additionally, our clock-hour based analyses of the extents revealed a significant correlation with functional parameters.

Previous studies demonstrated the common areas for early changes in hydroxychloroquine retinopathy, the inferior or temporal parafovea^4,17,18^. In the reports from Asian patients, the areas of most extensive damage or those of early changes were similar to those obtained from parafoveal cases^9,19^. However, RPE damage has not been separately analyzed in previous studies. The present study, with separate analyses on photoreceptor and RPE defects, showed that parafoveal RPE damage demonstrated a similar distribution to photoreceptor damage, more frequently in inferior and temporal locations, whereas pericentral RPE damage showed nasal or peripapillary areas as the most common or earliest changes, as demonstrated in Figs. 3 and 5. Accordingly, the area might be considered a “vulnerable zone” of RPE damage. This suggests cautious examination of the peripapillary areas for evaluation of the stage and progression of pericentral retinopathy, as the RPE damage, indicating progression to severe stage, frequently or initially occurring in the area.

This study also separated and compared the findings obtained using the two most common structural modalities used for screening and monitoring hydroxychloroquine retinopathy, OCT and FAF. In the comparison, the clock-hour extent obtained by OCT was greater than that obtained by FAF, suggesting a more sensitive detection of photoreceptor defects by OCT, whereas both modalities had comparable abilities to detect RPE damage. The discrepancy in photoreceptor defects on OCT and hyperautofluorescence on FAF may lead to differences in retinopathy severity between the modalities; for instance, the case judged as moderate on OCT images might be deemed early on FAF images. The subjective nature of determining FAF alterations and the low contrast between hyperautofluorescence and normal autofluorescence may partly explain the discrepancy in photoreceptor damage between OCT and FAF. This indicates that OCT, with sensitive detection ability, may be better for determining the extent of photoreceptor damage than FAF. In contrast, hypoautofluorescence due to RPE damage showed distinctive changes and contrast on FAF imaging, leading to almost similar sensitivities between OCT and FAF.

From the photographic examples presented in this study and the cases reported in previous studies, hydroxychloroquine retinopathy is characterized as ring-shaped lesions^4^, which functionally leads to ring scotoma^20^. Earlier cases with patchy or partial-ring lesions showed circumferentially dominant progression as the disease advances^8,9^, which suggests that the documentation of clock-hours may be useful for conveying topographic information as well as the extent of damage in patients with hydroxychloroquine retinopathy. This system has been widely used to describe the extent of retinal involvement in retinopathy of prematurity^21–23^, as the disease occurring at the junction of the vascular and avascular retina, is generally circumferentially oriented. From the circumferential pattern of damage and its progression in hydroxychloroquine retinopathy^9^, the extent may be described as clock hours in the retinopathy, for which we presented its usefulness for documentation of topography and extent of the disease.

Under the current classification, the distinction between early and moderate, based on 180°, is limited to providing detailed information about retinopathy extent, and the severe stage does not provide any information about the involved areas^7,9–12^. This may lead to wide variability in the perimetric parameters in each severity group as well as a discrepancy in the perimetric function between moderate and severe retinopathy (Figs. 5 and 6). For alternative classification scheme based on the involved areas, the photoreceptor defect areas on the ETDRS grid or other kinds of macular grid may be utilized for staging and functional correlation. However, neither grid can fully cover the areas with outer retinal defects in eyes with pericentral retinopathy, usually around the major vascular arcade. In contrast, the clock-hour extent method may be useful for detailed documentation of the degrees of structural damage, in both parafoveal and pericentral diseases. It is valuable for making functional correlations in eyes with hydroxychloroquine retinopathy, as in Fig. 4 showing a linear relationship between extent and parameters. However, to evaluate the extent of retinopathy in hydroxychloroquine retinopathy, radial OCT scans with at least six lines are required for clock-hour documentation. These scans are available from several types of commercially available OCT devices, although the number of scans varies among devices.

Functional disturbances in hydroxychloroquine retinopathy are visual field defects and their expansion, which eventually lead to vision loss in eyes with fovea-involving retinopathy. The documentation of the clock-hour extent of retinopathy may be useful in conjunction with the current classification for the functional correlation. However, this may be valid only until the defective areas become a complete ring, as eyes with full-ring lesions in our study, which involved all clock-hours, showed widely variable perimetric functions (Fig. 4). For eyes with full-ring lesions, the centripetal/centrifugal extent might be more useful for functional correlation and an indicator of disease severity, as the lesions subsequently show remarkable progression in the centripetal/centrifugal fashions^9^, although this requires further validation.

Several limitations should be considered when interpreting our findings. First, the retrospective nature of the study inevitably resulted in an intrinsic selection bias. All patients included in this study were of Asian ethnicity; therefore, most eyes showed pericentral involvement^15,24^, and a relatively small number of eyes with parafoveal retinopathy were included. These ethnic differences in the proportion of parafoveal and pericentral retinopathy may significantly affect the overall extent of photoreceptor and RPE defects in parafoveal and pericentral areas. Accordingly, we provided the data in all eyes and those with parafoveal and pericentral involvement, for the applicability of our data, regardless of the ethnic background of the population. Further, the proportion of eyes with severe retinopathy included in our study was relatively high, and we believe that this is associated with late diagnosis of pericentral retinopathy in the Asian population^15,24^. This may have a significant impact on the data of the extent of the retinopathy. Moreover, multifocal electroretinography (mfERG) results were available only for a few patients. Because mfERG can be used to topographically correlate functional defects with structural findings^25^, this should be evaluated in future studies as it may be valuable for understanding structure–function relationship in hydroxychloroquine retinopathy and also for the early detection of hydroxychloroquine retinopathy.

In summary, this study showed clock-hour topographic characteristics and extent of the photoreceptor and RPE damage in eyes with hydroxychloroquine retinopathy. Our study highlighted discrepancies in the extent of photoreceptor defects between FAF and OCT imaging and that in the topography between photoreceptors and RPE damage in eyes with pericentral retinopathy. Based on the significant correlation between the clock-hour extent of outer retinal damage and the perimetric parameters, the evaluation of hydroxychloroquine retinopathy according to clock-hours of the involved areas may be clinically useful in terms of structural documentation and functional correlation.

## Supplementary Information

Supplementary Figures.
