# Supplementary material for: Clock-hour topography and extent of outer retinal damage in hydroxychloroquine retinopathy
Source: Sci Rep. 2022 Jul 12;12:11809. doi: 10.1038/s41598-022-15217-3 (PMC9276819; doi:10.1038/s41598-022-15217-3)

Supplementary Materials

Supplementary Figure S1

Supplementary Figure S2

Supplementary Figure S1. Percentages of eyes with early retinopathy having photoreceptor defects on OCT and hyper- or hypoautofluorescence on fundus autofluorescence (FAF) in the parafoveal area among eyes with parafoveal involvement (left) and those on the pericentral area among those pericentrally involved (right).

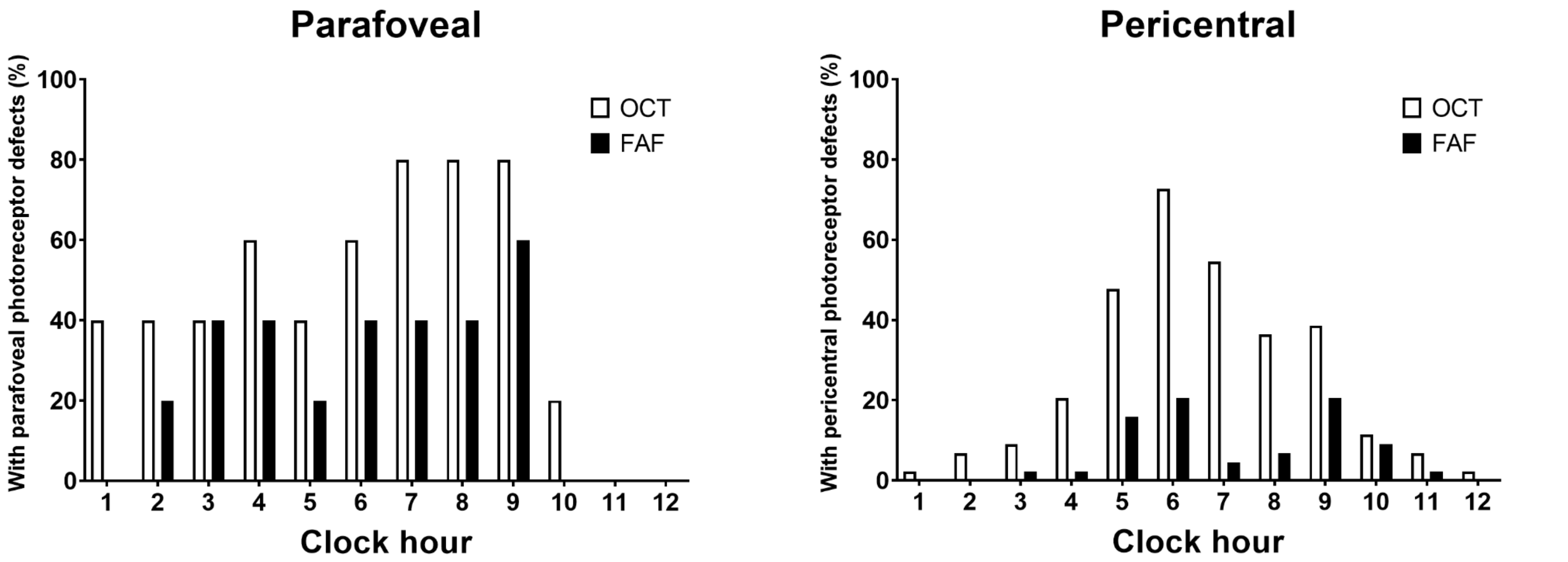

Supplementary Figure S2. Correlation between clock-hour extents of parafoveal retinal pigment epithelium (RPE) defects and mean deviation (MD) and pattern standard deviation (PSD) on the Humphrey 10-2 test (top) and that between those of pericentral RPE defects and MD, PSD, and visual field index on the 30-2 test (bottom).

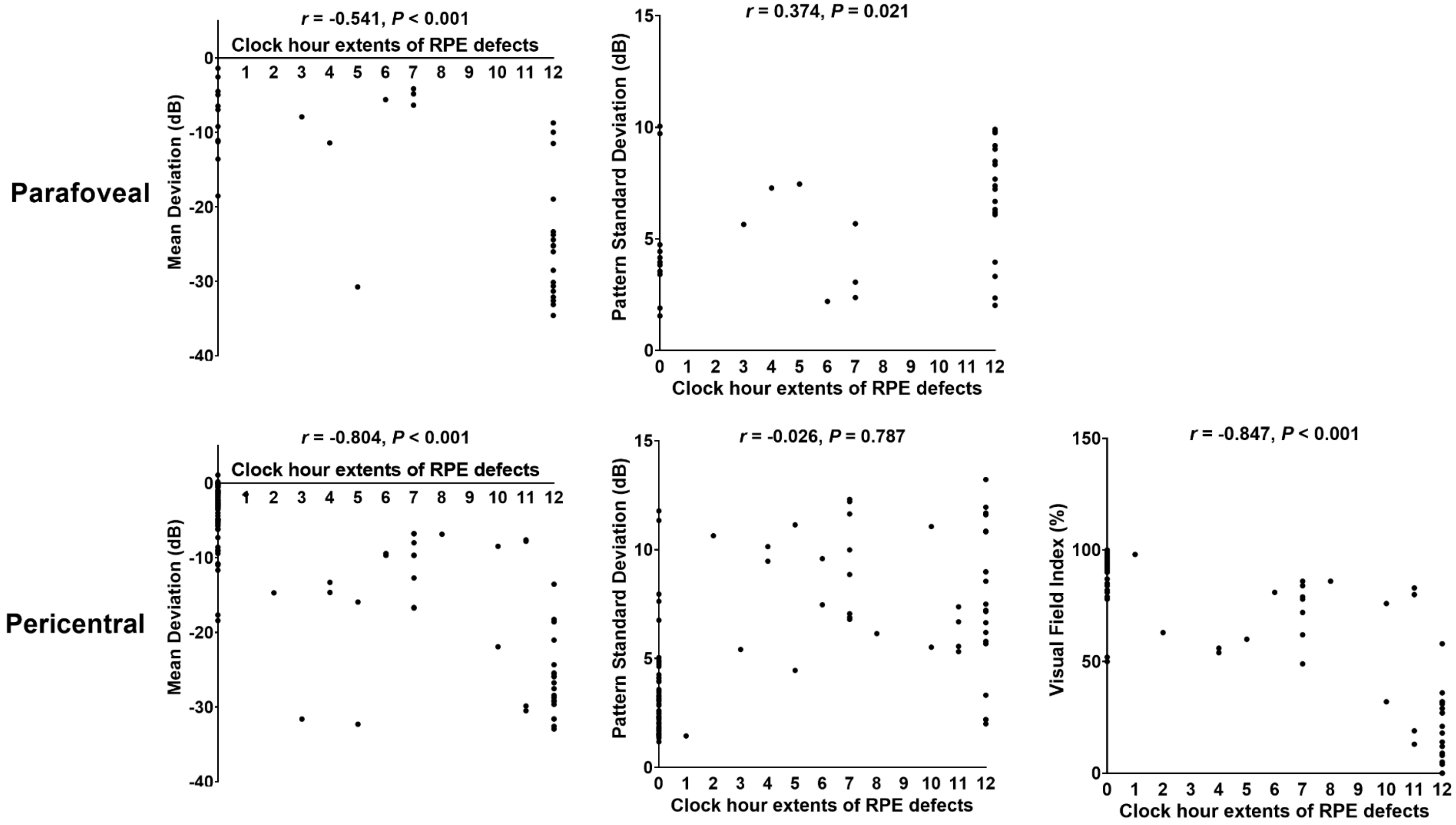

Supplement: Supplementary file 1 — Supplementary Figures. [file 41598_2022_15217_MOESM1_ESM.pdf]
